# Correctional nursing: a study protocol to develop an educational intervention to optimize nursing practice in a unique context

**DOI:** 10.1186/1748-5908-8-71

**Published:** 2013-06-21

**Authors:** Joan Almost, Wendy A Gifford, Diane Doran, Linda Ogilvie, Crystal Miller, Don N Rose, Mae Squires

**Affiliations:** 1 School of Nursing, Queen's University, 92 Barrie Street, Kingston, ON K7L 3N6, Canada; 2 School of Nursing, University of Ottawa, 451 Smyth Road, Ottawa, ON K1H 8M5, Canada; 3 Saint Elizabeth, 90 Allstate Parkway, Suite 300, Markham, ON L3R 6H3, Canada; 4 Lawrence S. Bloomberg Faculty of Nursing, University of Toronto, 155 College Street, Toronto, ON M5S 3H4, Canada; 5 Ministry of Community Safety and Correctional Services, Government of Ontario, 25 Grosvenor Street, 16th Floor, Toronto, ON M7A 1Y6, Canada; 6 Toronto South Detention Center, Transition Unit, Etobicoke, ON M8Z 4X8, Canada; 7 Daphne Cockwell School of Nursing, Ryerson University, 350 Victoria Street, Toronto, ON M5B 2K3, Canada; 8 Kingston General Hospital, 76 Stuart Street, Kingston, ON K7L 2V6, Canada

**Keywords:** Nurses, Nursing, Corrections, Prisons, Evidence-informed care, Education intervention, Learning needs, Work environments

## Abstract

**Background:**

Nurses are the primary healthcare providers in correctional facilities. A solid knowledge and expertise that includes the use of research evidence in clinical decision making is needed to optimize nursing practice and promote positive health outcomes within these settings. The institutional emphasis on custodial care within a heavily secured, regulated, and punitive environment presents unique contextual challenges for nursing practice. Subsequently, correctional nurses are not always able to obtain training or ongoing education that is required for broad scopes of practice. The purpose of the proposed study is to develop an educational intervention for correctional nurses to support the provision of evidence-informed care.

**Methods:**

A two-phase mixed methods research design will be used. The setting will be three provincial correctional facilities. Phase one will focus on identifying nurses’ scope of practice and practice needs, describing work environment characteristics that support evidence-informed practice and developing the intervention. Semi-structured interviews will be completed with nurses and nurse managers. To facilitate priorities for the intervention, a Delphi process will be used to rank the learning needs identified by participants. Based on findings, an online intervention will be developed. Phase two will involve evaluating the acceptability and feasibility of the intervention to inform a future experimental design.

**Discussion:**

The context of provincial correctional facilities presents unique challenges for nurses’ provision of care. This study will generate information to address practice and learning needs specific to correctional nurses. Interventions tailored to barriers and supports within specific contexts are important to enable nurses to provide evidence-informed care.

## Background

Nurses are the primary healthcare providers in correctional facilities. The complex health needs of inmates require nurses to have specialized knowledge with strong assessment and clinical decision-making skills to care for a clientele who may desperately need healthcare, but may be manipulative and aggressive [1]. Responsibilities include elements of outpatient care, emergency nursing, psychiatric-mental health, occupational health, and community health [2]. Therefore, a solid knowledge and expertise in clinical decision making is needed to optimize nursing practice and scope of practice within these settings. However, heavy workloads, lack of time and insufficient staffing have led to correctional nurses not always being able to obtain the training or ongoing education that is required to provide evidence-informed care within their broad scopes of practice [3]. In addition, while the delivery of healthcare is important, there is an ongoing struggle to find the balance between prisoners’ healthcare needs and institutional emphasis on custodial care and security. The heavily secured, regulated, and punitive environment that exists in prisons presents unique contextual challenges for nurses to provide evidence-informed care. The purpose of the proposed study is to develop an educational intervention for correctional nurses to support the provision of evidence-informed care. The intervention will be tailored to meet the learning needs of correctional nurses working in Canadian provincial facilities and delivered within the unique context of their work environment. Results will be used to inform a more rigorous evaluation of the intervention and impact on inmate and process outcomes.

This project will be guided by four objectives: describe the scope of nursing practice in provincial correctional facilities; identify correctional nurses learning needs; describe work environment characteristics that support evidence-informed care; and develop and evaluate the acceptability and feasibility of an online education intervention to support evidence-informed care within the unique context of provincial correctional facilities.

### Scope of practice

A broad scope of practice has been identified as an important issue in the work environments of correctional nurses. However, in a previous study, 40% of nurses in provincial correctional facilities reported not being able to practice at their full scope [3]. In addition, nurses reported feeling unprepared in their roles within a work environment that has limited resources to meet their learning needs [3]. A review of the literature found little research on the scope of practice of correctional nurses. While there is no clear definition, scope of practice refers to standards of practice or professional competencies, legal base of practice, or components of the clinical parameters of practice [4]. It is important to understand how correctional nurses describe their scope of practice and develop strategies that will enable their skills to be better utilized [5], including factors that address working conditions and organizational characteristics [6].

### Work environment characteristics and ongoing learning needs

The ability to work to full scope is facilitated through a variety of mechanisms, such as legislation and regulation. More importantly continuing competence through the support of continuing and additional education opportunities is an important facilitator [7]. Formal and informal learning can contribute to the nurses’ progression from novice to expert, enabling nurses to respond to constantly changing technologies and systems, as well as specific client and career needs. Thus, employers can have a significant impact on a nurse’s ability to practice to a full scope through policy, resources, and access to education [8]. There is limited research on work environment characteristics that act as barriers and/or facilitators to correctional nurses’ ability to work to their full scope and ongoing learning. The findings from our previous research with correctional nurses suggest that barriers exist related to the unique environment of the correctional setting, heavy workloads, inadequate staffing, lack of resources, and lack of policies [3]. However, further work is needed to fully understand the nature of these barriers and how they constrain ongoing learning.

### Significance of the study

Ensuring that nurses are able to work to their full scope is an important strategy that is crucial to attracting and retaining a skilled nursing workforce and providing quality care. The issue of retention and safety is a key concern for policy makers. This study aims to address the retention of correctional nurses by creating opportunities for correctional nurses to have their practice needs identified and their learning needs met. This study will generate information on priority issues identified by both the Ministry of Health and Long-Term Care and the Ministry of Community Safety and Correctional Services with a particular emphasis on innovative solutions to address learning needs and work to develop supportive environments specific to correctional nurses.

## Methods

A mixed methods research design with two phases will be used. The setting will be three provincial correctional facilities in Ontario that have access to the Ontario Telemedicine Network. This provides the opportunity and infrastructure for the delivery of education programs to take place via two-way videoconferencing systems and enhances opportunities for alternative forms of professional development for healthcare providers. Eligible study participants will be all registered nurses (RNs), registered practical nurses (RPNs), and healthcare managers (HCMs) working in these three facilities. The first phase of the study will focus on describing the scope of nursing practice in provincial correctional settings (objective one), identifying correctional nurses learning needs (objective two), and the work environment characteristics that support or impede evidence-informed care (objective three). Phase one will conclude with the development of an educational intervention designed to address the learning needs identified by the nurses (objective four). Phase two will evaluate the acceptability and feasibility of the intervention (objective four).

### Phase one: assessment and development of intervention

#### Setting

There are 30 provincial correctional facilities, detention centers, and jails across Ontario, with five of these facilities having access to the Ontario Telemedicine Network. To obtain a representative sample, three of the five facilities will be selected based on facility size and geographic location.

#### Sample

The sample will consist of all RNs, RPNs, and HCMs working full-time, part-time, or casual in the three facilities. There are approximately 95 nurses and three healthcare managers who are expected to be eligible to participate.

#### Approach to data collection

Both qualitative and quantitative approaches will be used to provide a complete picture, considering multiple perspectives [9], and triangulation of data [10]. Data collection will involve semi-structured interviews, Delphi process, and pre-intervention survey.

#### Semi-structured interviews

Approximately 18 to 21 face-to-face semi-structured interviews (five to six per site) will be conducted to obtain information about scope of nursing practice, learning needs, and work environment characteristics that act as barriers and facilitators. It is difficult to determine *a priori* the exact number of nurses who will be interviewed because sampling will be based on data saturation (*i.e.*, extent to which the research questions are addressed). The Nursing Role Effectiveness Model [11] will guide the interview process. The Nursing Role Effectiveness Model was developed by Irvine *et al.*[11] to identify the contribution of nurses’ roles to outcome achievement. The model is based on the structure-process-outcome model of quality care [12]. Interview questions (see Additional file 1) will explore the extent to which nurses are able to fully engage in role activities, the extent to which structural variables constrain or enhance practice, and the impact that nurses’ scope of practice has on nurse outcomes.

#### Delphi process

A Delphi process will be used to rank the learning needs identified by interview participants to facilitate priorities for the intervention. Interview participants will be invited to participate in two or three rounds of a Delphi process consisting first of ranking the importance of specific learning needs. In the second round the results of the rankings will be fed back to the participants and they will once again be invited to rank their learning needs, now with the knowledge from others’ rankings. The rounds will be repeated until the percent agreement among raters stabilizes. Priority learning needs will be selected based on those that achieve 80% consensus.

#### Pre-intervention survey

To describe the work environment characteristics that support evidence-informed care and augment description of the units, participants will be asked to complete two validated instruments:

1. Alberta Context Tool (ACT) [13]: Organizational context is defined as the environment or setting in which a proposed change is to be implemented [14]. The ACT measures features of the organizational context conducive to evidence-informed care with a focus on leadership practice, structures, and culture that support nursing practice. The scale consists of 56 items reflecting the following eight conceptual dimensions: culture (six items), leadership (six items), opportunity for evaluation (six items), social capital (six items), formal interactions (five items), informal interactions (seven items), structural/electronic resources (eleven items), and organizational slack (nine items). Scale items are scored on a five-point Likert frequency scale. In previous research, the Cronbach alpha for the sub-scales has ranged from 0.54 (electronic resources) to 0.91 (leadership) and evidence of construct validity has been demonstrated through an association between the 13 subscales and nurses’ research utilization [13].

2. Unit Technology [15]: The degree of complexity associated with nurses’ work (unit technology) will be measured by a 21-item questionnaire which incorporates the dimensions of uncertainty (ten items), instability (eight items), and variability (three items). Uncertainty describes the degree of difficulty and complexity of work, which is reflected in inmates’ illnesses and treatments, social-psychological nature of nursing care, and changes in tasks due to inmate’s conditions. Instability describes the fluctuation in nurses’ practice due to unpredictable changes arising from inmates’ health conditions. This is reflected in frequent nursing observations, multiple tests, technical equipment, time pressures, and frequent emergencies. Variability refers to unpredictability from variations among inmates and their nursing care needs, reflected by the variety of inmates’ problems, nursing care, and nurses’ decisions. In previous research, the reliability (Cronbach alphas ranging from 0.82 to 0.90) and validity have been tested in acute care, psychiatric and community health settings [16-18].

A demographic questionnaire will include questions about nurses’ age, experience, education, hours worked, hours of continuing education opportunities, and the nature of the continuing education opportunities. Participants will also be asked to complete a survey to evaluate their knowledge acquisition before and after the intervention. The content and questions will be based on the topic chosen by participants.

### Approach to data analysis

#### Interviews

Interviews will be audiotaped, transcribed verbatim, and analyzed using content analysis methodology. A structured process will be used to code the data. Two members of the research team will code the interview data. Inter-rater reliability will be determined based on a subset of four interviews, and will be repeated until satisfactory agreement among raters is achieved (*i.e.*, 80% agreement of coded data). Discrepancies in coding data will be resolved by consensus.

#### Delphi process

Frequencies and percentages will be used to summarize learning needs. Learning needs and preferences will be assessed by subpopulation, such as early career, late career, or previous work experience (*e.g.*, acute hospital, public health) before joining the provincial correction system.

#### Pre-intervention survey

Descriptive statistics (means, standard deviation, and frequencies) will be calculated for the survey data in order to describe the characteristics of the practice environment, nurses’ job characteristics, and the complexity of the work environment. Because several instruments will be used for the first time in the correctional setting, reliability analysis, using Cronbach’s alpha, will be conducted on the multi-item scales to determine the reliability of the measurement tools in this sample of nurses. Descriptive statistics will be used to describe the demographic characteristics of the participants during each data collection approach.

The findings from the interview, Delphi and pre-intervention survey will inform the development of the study intervention. These findings will provide information about nurses’ learning needs and awareness/preference of learning style, as well as factors that act as facilitators and barriers in meeting these learning needs. Information will also be obtained on preferred educational delivery methods such as independent online study or use of webinars. The educational intervention will then be implemented in the three facilities, with the goal of supporting nurses in their practice, creating a program that is feasible, and meeting the learning needs of correctional nurses.

#### Phase two intervention

The intervention will be developed using the principals of adult learning. A review conducted by Collins [19] identified several key principles of adult learning, including the level of current knowledge of an adult learner, their experience, and pre-existing skills in a particular area. This information will be used to help inform the refinement of the education program to meet the identified needs. Adult learners tend to be relevancy oriented, seeking information that they can directly apply, therefore it is important that the intervention be tailored to meet the practice environment of the learners [19]. Finally, research by Kolb and Fry [20] reinforces the importance of addressing the preferred learning style of adult learner.

#### Education

A skill and competencies upgrade training program is currently being delivered by a Canadian community healthcare organization to nurses working in federal correctional settings. As a result, they have produced an educational program that has already been developed, implemented, and tested. This program was developed in close collaboration with federal correctional services, but unlike this proposed initiative, was not based on a learning needs assessment. The program delivers education on various aspects of nursing practice relevant to the correctional environment. It incorporates both theory and practical components and consists of five modules: nursing process in assessing patients, fundamentals of nursing assessment, systems review, and pathophysiology; pharmacology; orientation to public health; addictions–clinical and pharmacological aspects; and standards of nursing documentation and legal aspects of nursing. Two additional modules on older offenders and age-related diseases and palliative care are in development. Educational materials include course material, relevant reference materials, learning activities, and a pre and post-test to evaluate participants’ knowledge acquisition. A course evaluation is completed by each participant, and a detailed instructor’s manual provides a review of each module, instructions on delivery methods, instructor’s notes, and overheads/ teaching aides. Evaluation of the program indicated that the majority of participants were ‘mostly’ to ‘very’ satisfied with the education and that a change in knowledge was seen between the pre and post scores for all five modules.

This suite of proven educational content will provide a framework for the intervention developed in this study. The federal correctional system is a similar environment, however, the inmate population is different from the provincial correctional system in terms of long-term versus short-term incarceration, characteristics, and behaviors. Therefore, the educational content will be tailored to the context and inmate population within the provincial correctional system.

#### Educational delivery

Initial assessment of the differences between the federal and provincial practice environments suggests that the current format of classroom learning will not be suitable for the provincial system where nurses’ workloads are high, and there is little time or resources to remove nurses from practice to engage in continuing education. Therefore, different formats for delivering the education will be explored. Current research evidence supports the use of e-learning as an effective knowledge transfer mechanism, which is based on adult learning principles and a constructivist approach to learning [21]. The paradigm of online teaching and learning shifts the focus from an authoritative/didactic approach to that of a facilitator or ‘guide-on-the-side’ where dynamic interactions with peers enhance learning. Successful online learning courses/programs engage learners via collaboration, reflection, and discussions/feedback, which help to increase retention rates and participant satisfaction [22].

The education will be made available through a web-based learning platform. This method will allow nurses to navigate through courses at their own pace and convenience and provide: consistency of training; accessible education 24 hours a day, seven days a week; an interactive discussion forum where topics can be posted and discussed; online activities such as case studies, reflection, and scenario-based learning; and instructors with the skills required to deliver online education. Depending on the learning needs of the nurses, e-learning strategies may need to be complemented with a practical component either through the use of simulation or a ‘hands on’ workshop to reinforce application of the knowledge and skills.

#### Intervention evaluation

In order to assess the feasibility of the intervention, data will be collected at the end of the intervention on response rate, attrition, perceived respondent burden, satisfaction, barriers and facilitators, and perceived utility of the intervention. In our previous study [3], correctional nurses completed a survey examining their work environment, job characteristics, and job satisfaction. In order to be able to compare the effects of the proposed intervention across time, participants will be asked to complete a similar survey at six months post-intervention, and their results will be compared to the previous survey which took place at the end of 2009. Participants will be asked to complete the Nursing Work Index-Revised, a global measure of job satisfaction and questions about job characteristics:

1. Nursing Work Index-Revised (NWI-R): Organizational attributes of the work setting will be measured by the Nursing Work Index-Revised [23]. Three subscales are derived from the NWI-R to measure organizational attributes noted in the literature as characterizing an environment supportive of professional nursing practice: autonomy, control over the work environment, and relationships with physicians. A fourth subscale is created from items in the previous three subscales to measure organizational support. Items are rated on a four-point Likert scale (strongly agree to strongly disagree) which are summed and averaged to yield the subscales. An overall NWI-R score is created by summing and averaging the subscales. A higher score indicates an environment that is supportive of professional nursing practice. The NWI-R has been used in multiple studies and has consistently demonstrated acceptable internal consistency reliability [23,24].

2. Job satisfaction: A two-item global measure of job satisfaction will be used. Using a four-point Likert scale (very dissatisfied to very satisfied), respondents are asked to rate their overall level of satisfaction with their present job and being a nurse (independent of current job). A higher score indicates a high level of job satisfaction.

3. Job characteristics*:* The survey will also collect data on job characteristics, specifically number of hours worked, amount of overtime required, intent to leave position, quality of nursing care, and ability to practice to full scope of practice.

#### Approach to data analysis

Descriptive statistics will be used to describe the demographic characteristics of the participants; frequencies and percentages will be used to summarize the evaluation of the intervention program. Correlations and regression analysis will be used to describe the relationships between factors within the work environment and the selected outcomes (job satisfaction). Paired t-tests will be used to test for significant changes in knowledge acquisition in the pre/post surveys and in perceptions of the nursing work environment (NWI-R), job satisfaction, and job characteristics over time (survey in 2009 to survey completed at six months post-intervention).

## Discussion

The proposed project will address several key nursing health human resource issues, specifically recruitment, retention, nurse preparation, and job satisfaction in a previously understudied sector in correctional nursing. The study will also reveal important findings regarding the context of the work environment of correctional nurses, as well as barriers and supports to correctional nurses’ provision of evidence-informed care. Very little research has been conducted with correctional nurses. We will build upon our previous research [3] and continue to examine the specific needs and challenges of this population of nurses, another key nursing research priority identified by the Ministry of Health and Long-Term Care. Ensuring that nurses are able to work to their full scope of practice is an important strategy that is crucial to attracting and retaining a skilled nursing workforce.

## Competing interests

The authors declare that they have no competing interests.

## Authors’ contributions

JA and DD led the application for funding. All authors contributed to the development of the study and writing the grant application. All authors approved the final version. JA and WG adapted the original grant application to meet the requirements for publication.

## Supplementary Material

Additional file 1 Interview Questions. Click here for file
