# Supplementary material for: Correctional nursing: a study protocol to develop an educational intervention to optimize nursing practice in a unique context
Source: Implement Sci. 2013 Jun 21;8:71. doi: 10.1186/1748-5908-8-71 (PMC3691633; doi:10.1186/1748-5908-8-71)
Supplement: Additional file 1 — Interview Questions. [file 1748-5908-8-71-S1.doc]

**Additional File 1. Interview Questions**

**Questions exploring scope of practice:**

1. What is the difference between RNs and RPNs scope of practice in correctional nursing?
2. Do you feel you are practicing at/below/above your scope of practice in correctional nursing? Why or why not?
3. From your perspective what sorts of things get in the way or support you to practice to your full scope?
4. What resources/supports do you think would enable you to perform within your scope of practice as a RN or RPN in correctional nursing?
5. If you were to make recommendations regarding strategies to be able to practice to your full scope what would they be?
6. Have you been able to use the full range of your education, knowledge and skills as a nurse? Why or why not?

**Questions exploring context/work environment:**

1. Are there factors that act as a barrier in allowing you to practise to the full extent of your skills and knowledge?
2. What are some specific examples from your practice setting when you were able to use the full range of skills and knowledge associate with your role as a nurse? When you were not able to use the full range?
3. Please comment on the workload on your unit. How do you think the workload affects your nursing practice? Your practice needs?
4. How do you feel about the effectiveness of communication between nurses and other team members on your unit? What are the barriers to communication? What are the facilitators? How could communication be optimized?
5. Could you comment on the coordination of care on your unit? Who is involved? What is your role? How well does coordination of care work? How do you think it could be enhanced?

**Questions exploring ongoing learning:**

1. What are your learning needs and awareness/ preference of learning style?
2. What factors (personal, professional, organizational, contextual) act as barriers in allowing you to have your learning needs met?
3. What factors facilitate or help to meet your learning needs?
4. Would you prefer independent online study where you can select the modules or to attend a more didactic Webinar?
